# Evolving and Novel Applications of Artificial Intelligence in Thoracic Imaging

**DOI:** 10.3390/diagnostics14131456

**Published:** 2024-07-08

**Authors:** Jin Y. Chang, Mina S. Makary

**Affiliations:** 1 Department of Radiology, The Ohio State University College of Medicine, Columbus, OH 43210, USA; jinyoung.chang@osumc.edu; 2 Division of Vascular and Interventional Radiology, Department of Radiology, The Ohio State University Wexner Medical Center, Columbus, OH 43210, USA

**Keywords:** artificial intelligence (AI), deep learning (DL), machine learning (ML), thorax

## Abstract

The advent of artificial intelligence (AI) is revolutionizing medicine, particularly radiology. With the development of newer models, AI applications are demonstrating improved performance and versatile utility in the clinical setting. Thoracic imaging is an area of profound interest, given the prevalence of chest imaging and the significant health implications of thoracic diseases. This review aims to highlight the promising applications of AI within thoracic imaging. It examines the role of AI, including its contributions to improving diagnostic evaluation and interpretation, enhancing workflow, and aiding in invasive procedures. Next, it further highlights the current challenges and limitations faced by AI, such as the necessity of ‘big data’, ethical and legal considerations, and bias in representation. Lastly, it explores the potential directions for the application of AI in thoracic radiology.

## 1. Introduction

Radiology is playing an increasingly pivotal role in healthcare, with the utilization of medical imaging consistently rising each year [1,2]. In the field of thoracic radiology, chest radiography (CXR) remains one of the most frequently performed imaging studies in medicine [3]. Moreover, the expanding implementation of national lung cancer screening programs is expected to substantially increase the utilization of chest computed tomography (CT) [4]. This escalating demand is placing a significant burden on the workload of radiologists.

Artificial intelligence (AI) has emerged as a promising solution to address many of the current challenges confronting radiology. AI has undergone substantial advancements, from machine learning (ML), which enables algorithms to learn from data without explicit programming, to the more recent deep learning (DL)—a subcategory of machine learning that utilizes layers of neural networks [5]. These techniques are now beginning to influence all parts of modern society, including the fields of medicine and radiology. With the clinical significance of thoracic diseases and the availability of abundant data, AI-based tools are actively being developed for thoracic imaging applications [3]. The United States Food and Drug Administration (FDA) has cleared more than 80 AI products for various indications within thoracic radiology alone [6]. As these devices demonstrate their efficacy beyond controlled test settings and transition into clinical environments, understanding how AI is currently being applied has become crucial. This review aims to provide an overview of the significant ways in which AI is reshaping the practice of thoracic radiology, while also discussing limitations and exploring the future directions.

## 2. Applications of Artificial Intelligence in Thoracic Imaging

**Figure 1:**
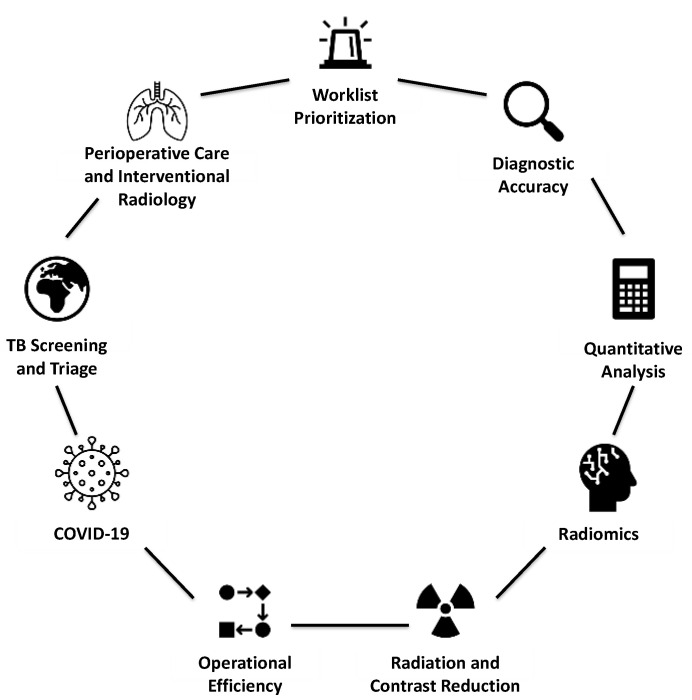
AI applications in thoracic imaging.

Summary of AI applications in thoracic imaging is presented in Figure 1.

### 2.1. Worklist Prioritization

AI was first used in thoracic radiology to detect CXRs with abnormalities and to prioritize them at the top of the reading worklist [7]. AI-based triage has shown promise in improving radiologist turnaround times, facilitating more timely diagnoses and management of urgent cases. Nam et al. developed a DL algorithm capable of accurately detecting 10 common abnormalities on CXR, with an area under the receiver operating characteristic curve (AUROC) ranging from 0.895 to 1.00 during external validation [8]. Prioritizing the picture archiving and communication system (PACS) worklist based on the algorithm’s results significantly reduced the time-to-report for critical and urgent cases compared to scenarios without the algorithm: 640.5 versus 3371.0 s and 1840.3 versus 2127.1 s, respectively (*p* < 0.01). Additionally, the interpretation time per CXR in AI-aided readings was significantly shorter compared to conventional reading sessions: 20.5 versus 23.5 s. Similarly, Baltruschat et al. introduced an AI-based worklist prioritization tool, which notably decreased the report turnaround time for all critical findings to 35–50 min, down from 80 min when employing a standard first-in, first-out processing approach [9]. In a simulation study, Annarumma et al. also demonstrated that a triaging system utilizing a DL algorithm reduced the median delay for critical examinations from 7.2 h to 43 min [10].

In addition to CXR, AI is also being explored for use as a triage tool in CT scans. Huang et al. developed a DL-based model capable of detecting pulmonary embolism in computed tomography pulmonary angiography (CTPA) scans, achieving an AUROC of 0.84 on the internal test set and 0.85 on an external dataset [11]. Furthermore, Hata et al. created a DL-based algorithm that identifies aortic dissection on non-contrast-enhanced CT images, achieving an area under the curve (AUC) of 0.940, with accuracy, sensitivity, and specificity comparable to those of radiologists [12]. As AI models begin to integrate into clinical setting as prioritization tools for various conditions, they will assist in identifying clinically important cases, thereby facilitating prompt diagnoses and treatments [13].

### 2.2. Diagnostic Accuracy

#### 2.2.1. Chest Radiography

A major application of AI in radiology is to enhance diagnostic accuracy in medical imaging. As a result, a significant portion of research of AI in thoracic imaging focuses on this topic [7]. While conventional computer-aided detection (CAD) has been used for decades, recent advancements in DL have significantly enhanced the capabilities of AI-based tools [14].

An effective AI model for lung nodules must not only accurately detect them, as they can be indicative of lung malignancies, but also minimize false positive results. Nam et al. developed a DL-based algorithm for detecting malignant pulmonary nodules on CXR [15]. The model achieved an AUROC between 0.92–0.99, outperforming 17 out of 18 physicians, while also exhibiting false positives rates ranging 0.02 to 0.34 per image. Additionally, all physicians showed improved nodule detection with the assistance of the DL algorithm. In a more recent study, Nam et al. demonstrated that the utilization of AI-based CAD significantly enhanced the detection of actionable (0.59% versus 0.25%, *p* = 0.008) and malignant nodules (0.15% versus 0.0%, *p* = 0.008) compared to not using AI in real-world health checkup participants [16].

In addition to detecting lung nodules, AI-based algorithms have also been developed for identifying other pulmonary abnormalities. Rajpurkar et al. developed a DL algorithm that was able to detect 14 different pathologies in CXR [17]. Compared to radiologists, the model achieved comparable level of performance on 11 pathologies, while radiologists achieved higher AUC on cardiomegaly, emphysema, and hiatal hernia. Similarly, Hwang et al. devised a DL algorithm capable of discerning between normal and abnormal CXR images, specifically targeting four major thoracic diseases [18]. The algorithm achieved a median AUC of 0.979, statistically outperforming non-radiology physicians, radiologists, and thoracic radiologists. Furthermore, the study demonstrated that physicians’ performance improved with the assistance of the DL algorithm. The development of newer deep learning AI algorithms may further enhance the accuracy of AI models in detecting pulmonary pathologies [19]. However, one limitation of DL-based CAD is that many studies have relied on isolated findings on CXRs, which may not accurately represent the multiple abnormalities that can present in real-world images [7].

The emergency department (ED) is an area where AI-based tools can provide crucial value in enhancing patient care. Hwang et al. demonstrated that a commercialized DL algorithm achieved an AUC of 0.95 in interpreting patient CXRs in the ED, showing higher sensitivity but lower specificity compared to radiology residents [20]. For the residents, the use of the algorithm resulted in an improvement in sensitivity from 65.6% to 73.4% (*p* = 0.003), albeit with a slight decrease in specificity from 98.1% to 94.3% (*p* < 0.001). Additionally, Huang et al. developed an AI model that generated reports based on CXRs from the ED and compared them with reports from a radiologist and teleradiologist [21]. When evaluated by six ED physicians using a Likert score, the reports generated by the AI model showed similar clinical accuracy and textual quality to that of radiologist reports, while demonstrating improved textual quality compared to teleradiologist reports.

In addition to pulmonary diseases, CXR can also detect non-pulmonary conditions. Pyrros et al. developed a DL model that identified patients with type 2 diabetes mellitus by integrating data from both CXR and electronic health records [22]. By analyzing adiposity features, the model achieved a diabetes detection AUC of 0.84 at a prevalence of 16%. Furthermore, the diagnostic utility of CXR in cardiovascular diseases such as heart failure, valvular heart disease, pulmonary hypertension, coronary artery disease, and atrial fibrillation is actively being studied [23,24].

Beyond improving diagnostic accuracy, studies are demonstrating that AI-based tools, when used as a second reader, also have the potential to enhance reader confidence and reduce reading time [25,26]. For instance, Ahn et al. reported a 10% reduction in reporting time for chest radiographs when using a commercially available AI engine compared to without AI (36.9 versus 40.8 s) [25]. Moreover, the implementation of AI can aid in efficiency by triaging and excluding normal chest X-rays, thereby reducing the workload for radiologists. In a stimulation study, Yoo et al. found that commercial AI triaging, which sorts and removes normal CXRs, led to a 40% decrease in workload [27]. Similarly, in an external evaluation, Plesner et al. found that a commercially available AI could autonomously report 28% of normal posteroanterior CXRs with a sensitivity of greater than 99% for detecting any abnormalities [28].

#### 2.2.2. Computed Tomography

The National Lung Screening Trial (NLST) and the Nelson trial have demonstrated the mortality-reducing benefits of low-dose chest CT screening for lung cancer in high-risk patients [29,30]. Consequently, the US Preventative Services Task Force (USPSTF) recommends annual screening for lung cancer with low-dose CT in adults who are at high risk [31].

DL algorithms have demonstrated high accuracy in detecting lung nodules, ranging from 82.2% to 97.6%, with corresponding AUC values of 0.87 to 0.98 [32]. Moreover, a systematic review by Ewals et al. demonstrated that with AI assistance, radiologists showed higher sensitivity and AUC in detecting lung nodules, although with slightly lower specificity [33]. Additionally, not limited to AI tools that directly identify abnormalities, AI-based image reconstruction model has shown to increase nodule detection rates, improve inter-reader agreement, and reduce reading time [34].

Furthermore, AI tools are capable of not only detecting lung nodules but also classifying them as benign or malignant. Ciompi et al. developed a DL system that classified nodules into four different types with an accuracy comparable to that of six physicians (69.6% versus 72.9%, respectively) [35]. Similarly, Ardila et al. achieved an AUC of 0.944 in predicting the risk of lung cancer with their DL algorithm [36]. The model, when no prior CT image was available, outperformed radiologists with an absolute reduction of 11% in false positives and 5% in false negatives. Recent advancements in AI models provide opportunities to expedite the diagnosis of malignant nodules, reduce unnecessary workup for benign nodules, and minimize inter-grader variability [37]. Moreover, Adams et al. demonstrated that integrating the AI risk score and Lung CT Reporting and Data System (Lung-RADS) in lung nodule management resulted in fewer follow-ups and reduced healthcare costs [38]. Additionally, a DL model by Mikhael et al. could predict lung cancer risk over the next 6 years with an AUROC of 0.75–0.81 based solely on a single low-dose CT scan, offering opportunities for early intervention and personalized patient care [39].

In addition to detecting and classifying lung nodules, AI tools can also aid in diagnosing other pulmonary diseases on CT scans. As previously described, DL-based AI models demonstrate high sensitivity and negative predictive value in detecting PE on CTPA scans [11,40]. Walsh et al. developed a DL algorithm for classifying fibrotic lung disease on CT images using existing diagnostic guidelines [41]. The model achieved an accuracy of 76.4% and 73.3% on different test datasets, outperforming 66% of thoracic radiologists. Similarly, Christe et al. developed a ML-based CAD system that classified idiopathic pulmonary fibrosis with accuracy comparable to two radiologists [42]. Furthermore, a DL tool developed by González et al. could detect chronic obstructive pulmonary disease (COPD) in CT scans with a C statistic of 0.856 [43]. The tool achieved accuracy rates of 51.1% and 29.4% for staging COPD in two different participant groups, as well as predicting the risk for acute respiratory disease events and mortality. Expanding beyond pulmonary pathologies, AI algorithms have been studied to assess cardiovascular health by evaluating coronary calcium score and predicting cardiovascular disease risk [44].

### 2.3. Quantitative Analysis

Quantitative analysis has emerged as a promising approach for identifying biomarkers associated with various pulmonary pathologies [45,46]. However, its widespread adoption in routine clinical practice has been hindered by the time-consuming nature of manual tasks [3]. Fortunately, advancements in AI technologies have enabled automatic segmentation of chest CT images with high accuracy and efficiency. For instance, Chassagnon et al. developed a DL algorithm capable of automatically assessing the extent of systemic sclerosis-related interstitial lung disease (ILD) on chest CT images [47]. The disease extent contouring by the algorithm was comparable to that of radiologists, with a median Dice similarity coefficient (DSC) ranging from 0.74 to 0.75, compared to a median DSC of 0.68 to 0.71 observed between radiologists. Additionally, the disease extent calculated by the algorithm correlated with the results of pulmonary function testing (PFT). Hasenstab et al. developed an automated DL algorithm for COPD staging by quantifying emphysema and air trapping [48]. The algorithm’s staging of COPD severity was matched spirometry-based Global Initiative for Chronic Obstructive Lung Disease (GOLD) staging, achieving an AUC of 0.86–0.96. Moreover, it successfully predicted disease progression (odds ratio, 1.50–2.67; *p* < 0.001) and mortality (hazard ratio, 2.23; *p* < 0.001). In addition to ILD and COPD, AI-based quantification analysis in segmentation has been studied for pulmonary lobes, airways, and lung nodules [49,50,51,52]. Furthermore, AI models can be employed to efficiently measure bronchial parameters, including in patients with cystic fibrosis [53,54]. Looking ahead, fully automated AI systems may play an essential role in preventive medicine and ‘opportunistic CT screening’ for patients [55].

The variability in patients’ characteristics, scanner types, acquisition techniques, and reconstruction protocols presents a challenge to the implementation of AI in clinical settings [14,56]. These variations impede the reproducibility and generalizability of quantitative image analysis, including radiomics [57]. Recent studies have focused on how AI models can reconstruct images to standardize quantitative analysis. Lee et al. developed a convolutional neural network (CNN)-based DL model capable of converting one CT kernel to another [58]. The converted images demonstrated a reduction in root mean square error (RMSE) compared to original images, with a mean reduction of 65.7%. Similar CT conversions models have also been developed for pulmonary nodules and ILD [59,60]. With the advent of newer models, the reduction in variability between images will facilitate AI research in quantitative imaging.

### 2.4. Radiomics

Radiomics is a field in radiology that aims to extract quantitative features from an image using algorithms [61]. Many of these features are invisible to the human eye and have been investigated as potential biomarkers, especially in thoracic oncology. Beig et al. utilized perinodular and intranodular radiomic features on noncontrast lung CT scans to differentiate between non-small cell lung cancer adenocarcinomas and benign granulomas [62]. Combining radiomic features in the analysis led to an increase in the AUROC from 0.75 to 0.80. Hawkins et al. found that radiomics applied to low-dose CT images can predict which nodules will become cancerous within 1 to 2 years [63]. The best-performing model was able to predict the emergence of cancer with accuracies of 80% (AUC 0.83) and 79% (AUC 0.75) at 1 and 2 years, respectively.

Beyond detecting lung cancer, radiomics are demonstrating their utility in characterizing and stratifying lung cancers for prognosis [64]. Fan et al. identified radiomic features that could discriminate between invasive adenocarcinoma and non-invasive lesions [65]. The radiomic signature achieved an accuracy ranging from 86.3% to 90.8% in the primary and validation cohorts. Similarly, Cherezov et al. demonstrated that a combination of heterogeneity and radiomic features discriminated between cancerous and non-cancerous nodules, achieving an AUROC of 0.85 and an accuracy of 81.64% [66]. Moreover, their study found that the analysis could also distinguish long- and short-term survivors of lung adenocarcinoma with an AUROC of 0.9 and an accuracy of 85%. Additionally, Wu et al. demonstrated how radiomics can be used as a non-invasive means to determine lung cancer histology by differentiating adenocarcinoma and squamous cell carcinoma, achieving an AUC of 0.72 [67].

Recent studies have investigated the association between radiomics and genomics, termed radiogenomics, to identify specific mutations and guiding treatment decisions [68]. Jia et al. demonstrated the identification of epidermal growth factor receptor (EGFR) mutation in lung adenocarcinoma using radiomic features, achieving an AUC of 0.802 [69]. Incorporating sex and smoking history further improved the model’s performance, with sensitivity and specificity reaching 60.6% and 85.1%, respectively. Rizzo et al. identified specific qualitative CT associations corresponding to various mutations in non-small cell lung cancer (NSCLC), including EGFR, Kirsten rat sarcoma viral oncogene homolog (K-RAS), and anaplastic lymphoma kinase gene (ALK) mutations [70]. Additionally, Trebeschi et al. demonstrated the potential of radiomics features as biomarkers for predicting the response to immunotherapy in NSCLC, achieving an AUC of 0.83. Their study found that the radiomic biomarkers were involved in cell cycle progression and mitosis, offering insights into the mechanism for the preferential response. By integrating genomic and phenotypic information found in imaging data, AI radiomics will expand our knowledge of tumor biology and promote precision medicine [71].

### 2.5. Radiation and Contrast Reduction

Image reconstruction techniques have been studied with the goal of improving image quality while reducing the radiation dose in CT scans. Iterative reconstruction (IR) algorithms have been introduced to replace conventional filtered back projection, with the goal of reducing image noise and optimizing radiation dose [72,73]. However, IR has its limitations, including over-smoothing and the production of altered image textures [14,73]. Recently, DL-based image reconstruction methods have demonstrated improvements in image denoising, texture improvement, and artifact reduction compared to conventional models at lower doses [74,75,76,77]. AI image reconstruction algorithms offer the promise of ultra-low-dose CT (0.07 mSv or 0.14 mSv) and potentially even photon-counting CT technology in the future [37,78,79]. Moreover, DL techniques can generate synthetic CT images from MRI data and enhance image quality by reducing metal artifacts [80,81]. Furthermore, DL-based grid-like algorithm has shown improvements in both image quality and dose reduction in CXR [82].

Iodinated contrast media, commonly used in CT imaging for diagnostic purposes, carry risks of allergic reactions and nephrotoxicity [83]. Recent advancements in AI now enable the creation of synthetic post-contrast images from non-contrast or low-dose post-contrast CT scans [84]. For instance, Choi et al. utilized a DL model to generate synthetic contrast-enhanced CT images from non-contrast chest CT scans [85]. These synthetic images significantly improved the detection of mediastinal lymph nodes, demonstrating a higher contrast-to-noise ratio (6.15 versus 0.74, *p* < 0.001) and enhanced lesion conspicuity (*p* ≤  0.001) compared to non-contrast CT scans. Additionally, Chun et al. produced a synthetic contrast-enhanced CT from non-contrast CT scans using DL, facilitating the delineation of cardiac substructures following breast radiation therapy [86].

### 2.6. Operational Efficiency

Not limited to diagnostic applications, AI can also be applied in operational tasks within radiology. From AI-assisted clinical decision support for imaging orders to improving automated speech recognition for report generation, AI has the capacity to aid in various aspects of the radiology workflow [87,88]. For instance, Chong et al. decreased the no-show rate for outpatient MRI from 19.3% to 15.9% by using an ML-based model to identify patients at high-risk of not showing up and implementing a telephone reminder system [89].

One notable AI application in thoracic imaging is its use in the follow-up of lung nodules. Pannu et al. showed that the rate of appropriate follow-up for incidental lung nodules remains low, at only 43.2%, representing a missed opportunity for early lung cancer detection [90]. To address this issue, natural language processing (NLP) is being explored as a means to efficiently identify reports mentioning pulmonary nodules and flag them for appropriate follow-up [91]. Desai et al. implemented an NLP algorithm that not only increased the rate of follow-ups completed on time from 64.5% to 84.3% but also achieved 87% agreement between primary care providers and radiologists on the follow-up plan [92]. Similarly, Abbasi et al. developed an AI model for identifying radiology reports containing recommendations for additional imaging, with the external validation set demonstrating 99% accuracy and an F1 score of 95.2% [93]. As the use of AI in non-interpretative areas continues to expand and more tools are integrated, AI is anticipated to play an increasing role in the radiology workflow.

### 2.7. COVID-19

The COVID-19 pandemic accelerated research into AI applications in thoracic imaging, which can be crucial for detecting COVID-19, assessing disease severity, and predicting prognosis [94]. While reverse transcriptase-polymerase chain reaction (RT-PCR) remains the gold standard for diagnosing COVID-19, challenges such as kit availability, reliability, and turnaround time persist [95,96]. In a systematic review, Guidgar et al. found that AI applied to various chest imaging modalities, including CXR, CT, and ultrasound, aids in the early diagnosis of COVID-19 [97]. Additionally, AI applications in chest CT facilitate the quantification and monitoring of COVID-19 progression [98,99]. Furthermore, AI in chest imaging provides prognostic information, including predicting the development of acute respiratory distress syndrome (ARDS), the need for oxygen therapy or intubation, and hospital stay duration or adverse outcomes [100,101,102,103,104]. In a meta-analysis conducted by Jia et al., AI models were found to distinguish COVID-19 from other pneumonias with a pooled AUC, sensitivity, and specificity of 0.96, 0.92, and 0.91, respectively [105].

Nevertheless, there remain limitations in the practical application of AI tools within the current clinical workflow. A systematic review by Roberts et al. revealed methodological flaws or a risk of underlying bias in machine learning models for detecting and prognosticating COVID-19 [106]. Additionally, these models require validation through prospective real-time studies. Sun et al. evaluated the real-time performance of an interpretable AI model for detecting COVID-19 on CXR, finding that the model had an AUC of 0.70 and underperformed compared to radiologists [107]. Furthermore, it is essential to address issues regarding the generalizability and applicability of AI models [108]. Continued research into AI for COVID-19 will be crucial in establishing the groundwork for preparing and managing potential resurgences of COVID-19 or other public health outbreaks in the future.

### 2.8. Tuberculosis Screening and Triage

Although tuberculosis (TB) is relatively rare in the United States, it was the leading global cause of death from a single infectious pathogen before COVID-19. In 2019, an estimated 10 million people developed active TB, resulting in 1.4 million deaths, with developing countries in Africa and Southeast Asia being disproportionately affected [109]. The World Health Organization (WHO) endorses the use of CXR both as a triage and as a screening tool for individuals at high risk of TB [110]. However, despite the high sensitivity and specificity of CXR for detecting pulmonary TB, its utility is limited by the availability of trained radiologists, particularly in countries with high prevalence [14,111].

AI for CAD has emerged as a promising solution to overcome existing limitations. While traditional machine learning techniques utilizing various features, such as texture analysis, for CAD were developed earlier, the advent of DL has significantly enhanced TB detection [3,112]. Hwang et al. developed a DL algorithm that achieved classification and localization performance with an AUROC ranging from 0.973 to 1.000, outperforming physicians, including thoracic radiologists, whose AUROC ranged from 0.746 to 0.971 [113]. Further studies have demonstrated that DL-based algorithms can now assess the disease activity, monitor post-treatment changes, and accurately identify active TB [114,115]. Additionally, Sethanan et al. developed a web-based application capable of classifying TB based on drug resistance from a single CXR [116]. In response to these advancements, the WHO, in 2020, recommended the use of CAD programs as an alternative to human readers for the screening and triage of TB [111]. Even when not used as stand-alone tools, AI may reduce reading time and improve accuracy when used as a second reader [117,118]. Although DL-based algorithms are currently being validated in real-world settings and there are concerns about increased false positive rates, they hold the potential to become a crucial tool in the global effort to eliminate TB [7,117,119].

### 2.9. Perioperative Care and Interventional Radiology

Imaging plays an essential role in medical procedures and perioperative tasks. Recent developments have increasingly highlighted the utility of AI in preoperative evaluations, extending beyond its established applications such as diagnosing, prognosing, and staging lung cancer [48]. One notable application of AI is its role in preoperative risk assessment. Given the high morbidity rates associated with thoracic surgeries, accurately evaluating individual patient risks before the procedure is crucial [120]. ML-based algorithms are demonstrating greater accuracy in assessing associated risks compared to conventional risk indexes [121,122,123]. Additionally, Chang et al. incorporated an ML model to predict the feasibility of immediate weaning from the ventilator for patients following lung resection [124]. Furthermore, Li et al. created a DL-based 3D reconstruction system capable of visualizing pulmonary anatomic structures, including segmental bronchi, arteries, and veins, with high accuracy [125]. Toggweiler et al. developed a fully automated DL model for planning transcatheter aortic valve replacement (TAVR) using CT imaging, achieving correlation coefficients of 0.95–0.97 for annular perimeter/area and ascending aorta [126]. As AI models continue to improve in accuracy and become more individualized, they will significantly aid in decision-making and planning for wide range of medical interventions.

Furthermore, AI offers various utilities directly related to interventional radiology procedures. Too et al. conducted a retrospective study on a DL model capable of generating needle trajectories for transthoracic lung biopsy [127]. Among the proposed trajectories generated by the software, 85.3% were found to be feasible, with 82% matching the actual path. Similarly, Kisting et al. developed an ML-based algorithm to evaluate pathways for percutaneous lung biopsies [128]. Their model had a correlation of 0.86 with physicians and demonstrated high sensitivity and specificity for safe and unsafe pathways (94.4% and 82.4%, respectively).

Image fusion, which integrates pre-procedural 3D anatomic data with real-time 2D fluoroscopy, offers improved precision during biopsies and ablations [129]. This technique involves incorporating AI model into virtual and augmented reality platforms for automatic landmark recognition, enhancing localization accuracy and efficiency [130]. Moreover, DL-based methods are being applied to generate digital subtraction angiography (DSA), enabling vessel extraction from a single live image without the use of a mask image, thus mitigating image artifacts [131]. With some studies already showcasing the feasibility of autonomous robotic surgeries, there is potential for further integration of AI to augment various procedures in the future [132,133].

**Figure 2:**
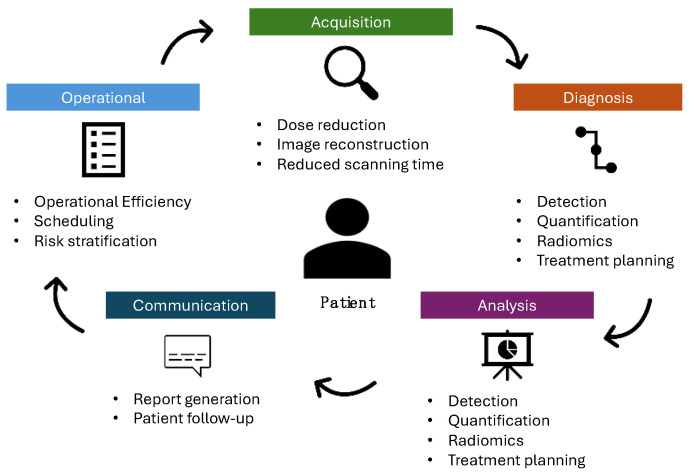
Representation of AI workflow in thoracic radiology.

Figure 2 illustrates how AI can contribute to the radiology workflow.

## 3. Limitations of AI

**Figure 3:**
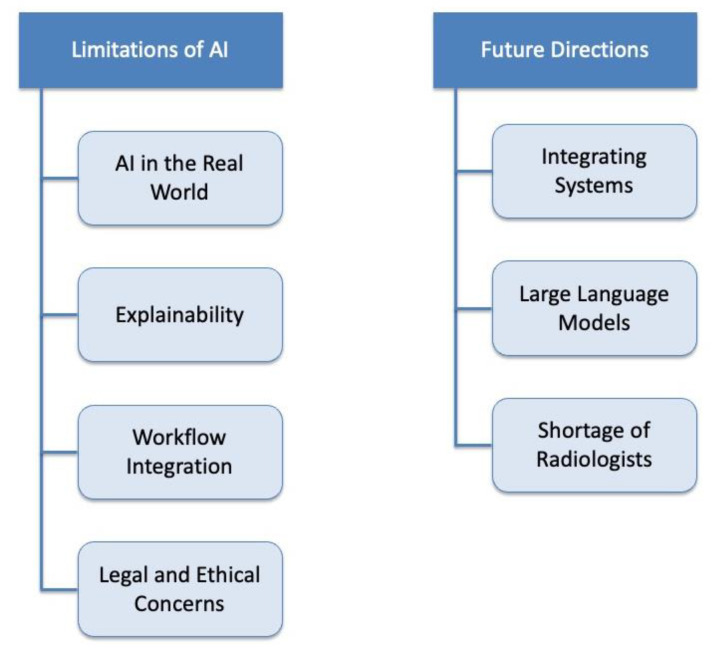
Limitations and future directions for AI applications in thoracic imaging.

An overview of the limitations and future directions for AI applications is described in Figure 3.

Table 1 presents an overview of the benefits and limitations of AI applications in thoracic radiology.

### 3.1. AI in the Real World

While AI algorithms have proven their efficacy in controlled test environments, achieving comparable performance in the real-world settings is paramount. The data used for training often exhibit a narrow distribution of variables and higher disease prevalence, potentially leading to ‘covariate shift’ or ‘probability shift’ [134]. Studies have found thoracic AI model to underperform in real-world practice and to have limited applicability [135,136,137]. Keane and Topol describe the concept of the ‘AI Chasm’, emphasizing the importance of developing an algorithm that will generalize across various populations and imaging studies rather than one that works well on a small dataset from a specific population [138]. One study found that the majority of studies on AI algorithms for diagnosis lacked external validation and design features necessary for testing in real-world scenarios [139]. Moreover, selection bias can manifest with any data set that fails to accurately represent the whole population [140]. Seyyed-Kalantari et al. found that AI algorithms trained using three large public CXR datasets displayed bias by underdiagnosing patients in underserved populations [141].

A primary cause and potential solution to this issue lie in the need for ‘big data’. Research on AI algorithms, particularly deep learning, requires access to high-quality, labeled, and publicly available data, which can be exceedingly challenging. Most data are not accessible for research, and even if they are accessible, the data is often not curated, organized, anonymized, annotated, or linked to the ‘ground truth’ [142]. Additionally, high-dimensional data are required to avoid ‘overfitting’, a phenomenon in which the algorithm fails to adequately perform on new data or different samples by learning the noise in addition to the actual data [143].

Numerous efforts are underway to overcome these obstacles, such as the de-identification process for protected health information (PHI) [144]. Natural language processing techniques are commonly employed to efficiently extract labels from radiology reports, but the generated labels may be inaccurate and prone to bias [145]. Innovative data-gathering methods that preserve privacy, such as federated learning, a method to transfer multiple decentralized data, or patient-mediated data sharing, where the patient contribute their own data for research, are actively being researched [142,146]. For instance, by utilizing federated learning, Dayan et al. developed a model that demonstrated a 16% improvement in average AUC across 20 participating sites and a 38% increase in generalizability for predicting outcomes in patients with COVID-19 [146]. Preprocessing techniques such as cross-population training and testing have been studied to mitigate biases such as sampling and confounding bias in training data [147,148]. Multicenter and international collaboration will be essential for the widespread application of AI in healthcare.

### 3.2. Explainability

One issue with AI algorithms, especially deep learning, is the explainability issue, or the ‘black box’ phenomenon, which describes the lack of transparency in how the algorithm makes decisions. The lack of understanding of how AI algorithms work and process data prevents acceptance and trust in the system [149]. Furthermore, the implementation of AI in this manner could significantly impact the training of new radiologists, as they might overly rely on the AI algorithm without meaningful interactions to develop their image interpretation skills [143]. While techniques such as saliency maps and feature visualization have been developed to address this concern, these methods may be insufficient for complex tasks [150]. Continued efforts to develop explainable AI models are needed to help gain approval from radiologists and integrate them into clinical practice.

### 3.3. Workflow Integration

Translating research findings into daily clinical practice is a crucial step in the application of AI. For AI assistance to enhance efficiency, seamless integration with minimal disruption is necessary. Additionally, the AI algorithm must display high performance in the targeted clinical cohort. Gaube et al. demonstrated that diagnostic accuracy significantly worsened when physicians received inaccurate advice [151].

Integrating the AI algorithm with PACS and electronic health records (EMR) is a necessary step to enhance operational efficiency. Moreover, AI models that can incorporate clinical context will improve decision-making. Huang et al. found that combining imaging with clinical data from the EMR led to increased accuracy and AUROC compared to single-modality models [152].

Most importantly, successful integration of medical AI relies heavily on support from radiologists. A study found that radiologists rated advice as lower quality when it came from an AI system [151]. Efforts to increase physician understanding of AI and its limitation are pivotal for further AI advancements [150]. Given the capacity of AI for self-learning, establishing a feedback system will be crucial for tailoring AI models to meet the diverse needs of all stakeholders. Demonstrating the clinical utility of AI through more clinical trials and observational studies is imperative, as improved diagnostic accuracy may not always translate to better patient outcomes [14,153]. Topol highlights the idea that metrics such as ROC or AUC are not necessarily indicative of clinical utility and do not demonstrate clinical efficacy [154]. High-quality evidence that determines the clinical impact of AI in healthcare, particularly focusing on the benefits for patients, is needed.

Furthermore, discussing reimbursement for AI model is vital for its widespread adoption [68]. Reimbursing AI technology separately should be considered if the application demonstrates clinical utility, such as improved diagnostic performance or providing new diagnostic information [155]. Reimbursement frameworks that incorporate all stakeholders, as well as propose the sustainable implementation of AI with high value, are being studied [155,156].

### 3.4. Legal and Ethical Concerns

One consideration with AI in radiology is data ethics. It is essential to address all aspects of data, including its collection and usage, data ownership, and protection [157]. Ongoing legislative efforts aimed at safeguarding patient privacy while facilitating innovative AI research will be pivotal [158].

Another issue is determining liability when utilizing AI model. It is essential to clarify responsibility for adverse outcomes, whether it lies with the manufacturer of the device, the hospital or department procuring the tool, or the physician utilizing the AI model [140]. Ambiguities persist, for instance in scenarios where AI triage model fails to detect a critical finding, resulting in an adverse event for the patient [143].

Implementing AI systems demands significant resources and expertise, which smaller hospitals and resource-limited countries may lack the capacity to accommodate [140]. Efforts to mitigate disparities in access to AI and its potential benefits are crucial. Additionally, resource-poor populations may be more vulnerable to automation bias, relying heavily on AI programs that could result in errors of omission and commission [157]. Equally vital to the development and implementation of AI systems is the promotion of safe and ethical practices for AI in healthcare.

## 4. Future Directions

While AI holds promise as a potential revolution in radiology, it remains in its early stages of development and requires ongoing validation. However, AI will continue to evolve with the advancements in data collection, algorithm development, and computational power. Initially focusing on non-diagnostic tasks such as worklist prioritization and operational workflow, AI will demonstrate its utility in the clinical setting. Regarding diagnosis, AI will greatly increase efficiency in detecting common diseases, while complex tasks will likely still remain the responsibility of radiologists [159].

It is crucial to recognize that the role of AI is not to replace radiologists but to assist them. Currently, the majority of AI algorithms are trained to excel at a single specific task. However, in the future, systems will have the capability to integrate multiple functions, such as image reconstruction, detection, and report generation, to significantly enhance lung cancer screening [160].

Large language models (LLMs), such as the ‘generative pre-trained transformer’ (GPT)-based models developed by OpenAI, are specifically designed to comprehend and generate text that closely resembles human language [161]. GPT models, based on DL principles, are gaining significant attention for their ability to generate output by learning from textual instructions, often requiring minimal training data [162]. Research has already been conducted on using GPT models to extract information from thoracic imaging reports [163,164]. In a study by Fink et al., GPT-4, the fourth version of GPT models, demonstrated high performance in extracting lesion parameters (98.6%), identifying metastatic disease (98.1%), and generating labels for oncologic progression (F1 score of 0.96) from CT reports on lung cancer [164]. Adams et al. employed GPT-4 as a tool to convert free-text radiology reports into structured templates for standardization [165]. Furthermore, Zaki et al. demonstrated the potential utility of LLMs in predicting the appropriate imaging study for a specific clinical presentation [161]. Applications of LLMs in healthcare can benefit both physicians and patients and span across multiple domains, including interventional radiology [162].

AI can significantly impact areas most affected by the shortage of radiologists, serving as a bridge to address resource limitation and barriers to health equity [166]. With AI technologies, healthcare providers can extend radiological services to underserved regions and populations, helping to reduce disparities in access to care.

## 5. Conclusions

Undoubtedly, AI will increasingly shape the future of radiology, necessitating a comprehensive understanding of its impact and limitations to adequately prepare for what lies ahead. Already, AI is influencing multiple aspects of thoracic radiology, ranging from detection to diagnosis and prognosis. It facilitates techniques such as quantitative imaging and radiomics, improving the characterization and understanding of pulmonary diseases. Additionally, AI streamlines operational workflow, enhances imaging safety, and enables faster diagnosis of critical cases. Furthermore, AI plays a critical role in global TB and COVID-19 management, while also contributing significantly to procedural planning and risk stratification. Despite challenges that remain in implementing AI in clinical practice, ongoing advancements promise continued improvement. The field of AI in thoracic radiology presents exciting opportunities revolutionize radiology practice and enhance patient outcomes worldwide.

## Figures and Tables

**Table 1:** Benefits and limitations of AI applications in thoracic radiology.

Applications of AI	Benefits	Limitations
Worklist Prioritization	High clinical impact Reduced turnaround time for critical cases Resource optimization	Liability Workflow integration
Diagnostic Accuracy	Improved diagnostic performance Reduced reading time Increased reader confidence	Explainability Lack of generalization Bias Interpretation errors
Quantitative Analysis	Efficiency Standardization	Data discrepancy Need for high quality data
Radiomics	Precision medicine Prognosis Thoracic oncology research	Standardization Workflow integration
Radiation and Contrast Reduction	Patient safety Enhanced image quality Cost saving	Workflow integration Algorithm accuracy
Operational Efficiency	Resource allocation Cost saving Less adverse risk	Workflow integration
COVID-19	Early diagnosis and monitoring	Real-world validation Lack of generalization
TB Screening and Triage	Address global health disparities and radiologist shortage Automated triage Early detection and treatment	Real-world validation False-positive rates
Perioperative Care and Interventional Radiology	Increased precision and accuracy Patient safety Risk assessment	Clinical integration Ethical concerns and liability

## Data Availability

No new data were created or analyzed in this study. Data sharing is not applicable to this article.
